# Caring for the child with intestinal failure on home parenteral nutrition: A scoping review

**DOI:** 10.1002/jpen.70028

**Published:** 2025-11-07

**Authors:** Mary Beth Hovda Davis, Valerie Boebel Toly, Erin Weber, Riad Rahhal, Ann Marie McCarthy

**Affiliations:** ^1^ University of Iowa College of Nursing Iowa City Iowa USA; ^2^ Case Western Reserve University Frances Payne Bolton School of Nursing Cleveland Ohio USA; ^3^ University of Iowa Stead Family Children's Hospital Iowa City Iowa USA; ^4^ College of Medicine Peoria University of Illinois Peoria Illinois USA

**Keywords:** caregivers, central venous catheters, child, intestinal failure, quality of life

## Abstract

**Background:**

Children with intestinal failure have significant long‐term medical needs that require continual complex procedures provided by the family caregiver in the home. This contributes to a high burden of care, leading to increased stress, anxiety, and depression. Understanding caregivers' perceptions of the homecare experience will augment healthcare providers' knowledge of how to prepare a family to provide care in the home setting.

**Methods:**

This study focused on the caregiving experience by examining the current literature. A scoping review was conducted using the Arksey and O'Malley methodology. Comprehensive searches on the caregiver of a child with intestinal failure receiving home parenteral nutrition were conducted in PubMed, Cumulative Index to Nursing and Allied Health Literature, Scopus, and Embase. Two reviewers screened the abstracts by title and abstract. One reviewer extracted then descriptively and thematically analyzed data to map the current evidence.

**Results:**

The initial search yielded 313 total articles. After inclusion and exclusion criteria were applied, a total of 15 relevant articles were included. Of the 15 studies, 5 studies described the caregiver's quality of life, 5 studies described caregiver's discharge preparedness, 3 studies discussed caregiver well‐being (stress, anxiety, and depression), and 2 studies described the caregiver daily experience of providing care.

**Conclusion:**

Preparing families to care for children with intestinal failure in the home may bolster caregiver preparedness but also impose a significant burden. Developing programs that offer optimal training, support, and interventions for caregivers can help alleviate stress and improve outcomes for both children and their families.

## INTRODUCTION

Children with intestinal failure who are dependent on home parenteral nutrition have significant long‐term medical needs, including the ongoing care of a central venous catheter (CVC). Maintaining a CVC outside of the hospital setting is a complex process that requires constant vigilance from the child's caregivers to avoid life‐threatening complications. Caregiver training, provided by healthcare professionals, requires the caregiver to develop critical thinking skills to understand not only the clinical concerns related to the underlying condition but also be capable of troubleshooting technical complications that can occur with home infusion devices.^1^, ^2^, ^3^

In the home setting, parents are expected to provide consistent, high‐quality care, 24 h a day, 7 days a week. Often, the responsibility of this care falls upon the mother or female caregiver of the child. In addition to providing all medical care for the child, the mother is also coordinating multiple medical appointments and therapies and often caring for other children.^4^ Although the family may qualify for home nursing hours through healthcare insurance, those hours are unlikely to be fulfilled by nurses with expertise or training in central line care and maintenance.^5^ Failure to provide high‐quality care in the home setting leads to CVC complications such as infection and the need for the child to be readmitted to the hospital.^6^

Findings from one study indicate that children discharged to home on home parenteral nutrition had at least one home parenteral nutrition–related complication within 30 days of discharge, and approximately 30% of children were readmitted within 30 days of discharge.^6^ Moreover, the odds for readmission were increased by 16% for every additional week of hospitalization during the initial hospitalization stay, with the average length of stay being slightly >80 days.^6^

Understanding caregivers' perceptions of the homecare experience will augment healthcare providers' knowledge of relevant topics needed to prepare a family to provide care in the home setting. Improving the preparation of families facing potential challenges at home can help keep the child safe in the home and out of the hospital. The purpose of this scoping literature review was to explore what is known regarding the experiences of caregivers providing medical care (such as home parenteral nutrition therapy via a CVC) for their child with intestinal failure in the home. The experience of caregiving can range from preparing caregivers to provide medical care in the home to how that care provision then impacts the caregiver's mental health and well‐being.

## METHODS

A scoping review methodological framework identified by Arksey and O'Malley was employed.^7^ To ensure that the appropriate scoping review stages were conducted, the Preferred Reporting Items for Systematic Reviews and Meta‐Analysis Extension for Scoping Reviews checklist was used.^8^ The Arksey and O'Malley framework consists of five stages: (1) identifying the research question, (2) identifying relevant studies, (3) selecting the studies, (4) charting the data, and (5) collating, summarizing, and reporting the results.^7^ The process is designed to be nonlinear, thus each stage allows for reflection and, if necessary, the ability to repeat steps to ensure that the literature is thoroughly reviewed.^7^

With the assistance of a health science librarian, the following databases were searched: United States National Library of Medicine (PubMed), Scopus, Cumulative Index to Nursing and Allied Health Literature, and Excerpta Medica Database (Embase). Search terms and results are reported in Table 1. In addition to searching electronic databases, reference lists from key articles were also searched to identify any additional references until a saturation of references was reached.

**Table 1:** Search strategies and databases.

Database	Search terms	Results (*n* = 313)
PubMed	(*intestinal failure* or *short gut* or *short bowel syndrome*) and (pediatric* or paediatric* or *child* or infant* or newborn* or *adolescent*) and (central line* or *central venous catheter* or *cvc*) and (*caregiver* or *caregiver burden* or parent* or *family*) and (*ambulatory* or *community* or *home*) and *home parenteral nutrition*	81
CINAHL	(*MH ‘Intestinal Failure’* or *intestinal failure* or *short gut* or *short bowel syndrome*) and (pediatric* or paediatric* or *child* or infant* or newborn* or *adolescent*) and (*MH ‘Central Venous Catheters+’* or ‘central line*’ or *central venous catheter* or *cvc*) and (*MH ‘Caregiver Burden’* or *caregiver* or *caregiver burden* or parent* or *family*) and (*ambulatory* or *community* or *home*) and (*home parenteral nutrition*) or *MH ‘Parenteral Nutrition Solutions’* or *MH ‘Total Parenteral Nutrition’*)	35
Scopus	TITLE‐ABS‐KEY (pediatric* or paediatric* or child* or infant* or newborn* or *adolescent*) and TITLE‐ABS‐KEY (‘central line*’ or ‘central venous catheter*’ or *cvc*) and TITLE‐ABS‐KEY (*caregiver* or parent* or *family*) and TITLE‐ABS‐KEY (*ambulatory* or *community* or *home*)	22
Embase	(‘*intestinal failure*’ or ‘*short gut*’ or ‘*short bowel syndrome*’) and (pediatric* or paediatric* or *child* or infant* or newborn* or *adolescent*) and (‘central line*’ or *‘central venous catheter’* or *cvc*) and (*caregiver* or *‘caregiver burden’* or parent* or *family*) and (*ambulatory* or *community* or *home*) and *“home parenteral nutrition”*	149
Citations from Key articles		26

*Note*: * is used at the end of the word to perform truncation therefore finding all variations of the wordstem.

Abbreviations: CINAHL, Cumulative Index to Nursing and Allied Health Literature; cvc, central venous catheter; MH, major heading; TITLE‐ABS‐KEY, article title, abstract, keywords.

The inclusion criteria were based on the purpose of the study and the researcher's previous review of the literature. The first inclusion criterion was that the study featured caregivers as subjects who care for children <18 years old with intestinal failure. The rationale for focusing on intestinal failure is the unique factors that put the child at higher risk for home infusion complications (eg, infection and CVC breakage).^9^, ^10^

The second inclusion criterion was that the study included children receiving home parenteral nutrition. The rationale for including home parenteral nutrition specifically is that home parenteral nutrition requires frequent access for home infusion and has a high risk for life‐threatening complications (eg, infection). The frequent access includes flushing the CVC, preparing and priming the tubing and home infusion equipment, a long infusion time (often 18–24 h), and a need to access the CVC to flush and lock before and after each infusion. Also, because of its high dextrose content, home parenteral nutrition is considered a high‐risk infusion, contributing to infection.^11^, ^12^, ^13^

Finally, a third inclusion criterion was published in the English language from January. 1, 2011 through January 29, 2024, as efforts to standardize central line care and maintenance practices and recognize associated infections were recommended by the Centers for Disease Control and Prevention (CDC) in 2011.^14^

The exclusion criteria for the scoping review were that the study (1) had a mixed population of children and adults with intestinal failure, (2) did not feature a caregiver providing care to a child, (3) did not exclusively include caregivers of children with intestinal failure, (4) included multiple patient diagnoses (eg, cancer), and (5) was a narrative review or conference abstract.

### Data extraction and synthesis

Abstracts were imported into a Rayyan database.^15^ Potential duplicates were then manually reviewed by the primary author MBHD, and 98 were removed. The initial review was conducted by two reviewers, MBHD and EW, who independently screened the abstracts using the inclusion and exclusion criteria. Any conflicts were reviewed in person and discussed to reach a resolution.

The resulting articles were imported into a new Rayyan database^15^ and independently reviewed by VBT and MBHD. Full copies of articles were retrieved and attached in the database to allow for access if clarification was required. Conflicts were resolved through in‐person discussion until consensus was achieved. Selected article data were abstracted and transcribed into an Excel database including the study author, intervention type, study population, study aims, methods, outcomes measures, and key results.

## RESULTS

**Figure 1:**
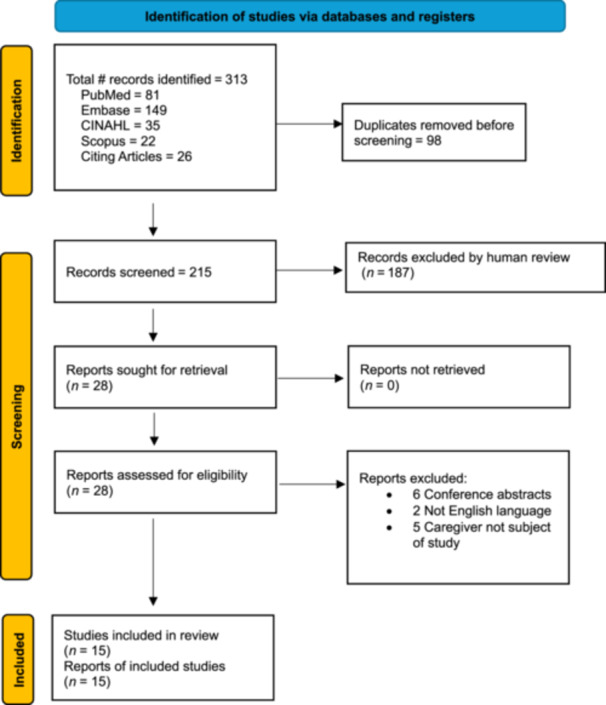
Preferred Reporting Items for Systematic Reviews and Meta‐Analysis 2020 flow diagram. CINAHL, Cumulative Index to Nursing and Allied Health Literature.

Search strategy terms and number of articles identified by each database are reported in Table 1. Figure 1 represents the screening process. The initial search yielded 313 total articles. Duplicates were removed manually, leaving 215 articles for primary screening. After inclusion and exclusion criteria were applied, 187 articles were excluded, leaving 28 articles for the secondary review. After the secondary review, a total of 15 relevant articles were included. Three studies were conducted using a qualitative or mixed methods methodology, eight were quantitative studies, and four were intervention studies. The results from the 15 articles are summarized in Table 2.

**Table 2:** Summary of included studies for scoping review.

Qualitative and mixed methods studies									
Author	Year of publication	Study location	Intervention type	Study population	Comparator	Aims of the study	Methodology	Outcome measures	Important results
Belza et al^28^	2022	Canada	None	13 caregivers of 11 children	None	Collect the experience of the caregivers caring for their child in the home to inform future development of support systems	Qualitative study: virtual focus groups	5 themes discovered: Caregiving is a 24/7 commitment Face constant risk of death Chronic illness creates difficult feelings and emotions Affects all aspects of family life Adapting and functioning as a family	Caregivers make significant sacrifices in their personal and family lives to meet the medical demands of their child. A crucial need is to identify resources and support to enable caregivers to provide high‐level care in the home
Neumann et al^21^	2024	USA	None	20 parents	None	Explore factors of care that impact the caregivers' QOL	Mixed‐methods pilot cross‐sectional survey	Caregiver QOL is negatively impacted by chronic sleep deprivation, psychological stressors, and access to support and resources QOL is less impacted by the logistical management of the child's medical needs	Teams should consider regular assessments of the caregiver's well‐being to identify unmet needs and addressing those needs of the caregivers. Future research is needed to identify interventions that can support caregivers
Wilcocks et al^27^	2022	USA		11 parents of children with SBS	19 comparison group; participants included parents of children with common GI complaints	Determine the stress, risk for poor mental health, and QOL of caregivers of children with SBS along with capturing the challenges experienced while providing medical care	Mixed methods	Parents of children with SBS have greater symptoms of stress and anxiety when compared with parents of children with GI complaints Complex treatment plans with limited respite care contributes to caregiver anxiety and hypervigilance, which significantly impacts the caregiver	Themes highlight the need to improve the quality of home healthcare, improve provider and patient communication, access to respite services, and the need to include mental health screening and related interventions specifically aimed at caregivers

Quantitative studies									
Author	Year of publication	Study location	Study population		Comparator	Aims of the study	Methods	Outcome measures	Important results
Belza et al^16^	2023	Canada	34 caregivers of children with IF		28 caregivers of healthy children	Determine caregiver burden of caregivers caring for children with IF receiving long‐term PN as it relates to their stress, anxiety, depression, and health‐related QOL	Cross‐sectional survey	Increased levels of stress for caregivers of children with IF compared with comparison parents (*P* < 0.01) Caregivers of children with IF demonstrated dec. health‐related QOL (*P* < 0.01)	Caring for a child with IF leads to a considerable burden to the caregivers demonstrated by high levels of stress, anxiety, and depression when compared with a healthy control group and other chronic pediatric patient populations
Belza et al^29^	2022	Canada	34 caregivers		None	Primary measure the time that caregivers spend providing medical care Secondary: identify factors that impact caregiver time, out‐of‐pocket expenses, and time and difficulty caregivers identify with providing care	Cross‐sectional study	29 h per week on average were spent providing informal care Alternate caregivers are important partners to compliment care burdens	The most significant time constraint included administering PN and caring for the CVC. The IF community needs to determine what services or resources can be implemented to provide adequate support to caregivers
Halsey et al^20^	2020	UK	14 children, 20 parents		None	Primary: Explore the emotional well‐being of caregiver Secondary: evaluate any connection between demographics and disease with emotional distress	Cross‐sectional survey	65% of parents had elevated anxiety; 35% were above clinical threshold 30% of parents had increased depression; 15% above clinical threshold Mean anxiety and depression score were higher than parents of children with neuromuscular disease*	Caregiver assessments for anxiety, depression, and stress along with subsequent treatments should be a part of routine care. Parental stress was higher than other published chronically ill populations
Neam et al^19^	2022	USA	117 families in an intestinal rehab program		Cohorts of families with healthy children and families of chronically ill children	Identify targets to focus interventions on to improve family health‐related QOL	Prospective descriptive study	Lowest QOL scores were found in caregivers of infants and the 5–7‐year‐old age groups IF families had lower QOL when compared with the healthy cohort* with similar scores to the chronically ill cohort Having a comorbidity and 4 or more inpatient admissions in the prior year were associated with lower QOL scores*	Families experience dec. QOL, which may improve as the patient ages. IF programs should address the family QOL in addition to just the patient
Pederiva et al^20^	2019	UK	30 families of children with SBS		Families of children with complex medical conditions	Evaluate QOL for families of children with SBS	Cross‐sectional study	SBS families had lower QOL when compared with families of chronically medically ill children Families with children over 5 years old felt a lack of information and communication and inclusion of the family	SBS impacts families' QOL, and future programs need to focus on the emotional and psychosocial functioning of the family and develop interventions to address these needs
Spagnuolo et al^26^	2013	Italy	16 families with IF		12 healthy control families	Assess the psychosocial well‐being of patients with IF and their families compared with healthy controls	Case control study	31% of caregivers demonstrated medium or high levels of anxiety Caregivers stated a lack of support from relatives and other care providers	Caregivers' QOL is negatively impacted by increased anxiety levels and reported lack of support outside of the family
van Oers et al^18^	2019	the Netherlands	62 mothers and fathers		Reference group of healthy children	Assess the caregiver QOL, depression, anxiety, distress, and everyday problems as compared with a healthy control	Cross‐sectional study	Mothers and fathers had higher distress scores than the comparison group Mothers had higher total problem scores than healthy control; father's scores were not different Caregivers were more likely to indicate wanting to talk to a professional than the healthy control group	No statistical differences in QOL between group, other than mothers having more depressive symptoms and fathers reporting distress with daily activities. Qualitative research may give more insight to the actual QOL. Structured parental psychosocial screening in clinical practice should be implemented to assess parental depression and distress
Witkowski et al^2^	2018	Brazil	27 caregivers and 17 children		None	Describe the outcomes of training family members for HPN directed to family members of children and adolescents participating in a multidisciplinary intestinal rehabilitation program of a tertiary public hospital	Descriptive cross‐sectional study	PN completed on time or delayed CRBSI rate CVC obstruction frequencies Accidental CVC dislodgement incidence Bleeding at insertion site Death	This study demonstrated that the discharge process for children and adolescents receiving PN could be feasible, safe, and effective. By training families to provide PN in the home, PN can be administered safely, but QOL for the caregiver was not assessed

Intervention studies									
Author	Year of publication	Study location	Intervention type	Study population	Comparator	Aims of the study	Methodology	Outcome measures	Important results
Drews et al^3^	2017	USA	CVC curriculum, including DVDs and impact on CLABSI	80 pediatric outpatients with IF	Retrospective data	Assess if an enhanced CVC curriculum would dec. CLABSI	Retrospective review of educational program	dec. CLABSI from 7.0 to 2.9 infections per 1000 CVC days (*P* = 0.001)	No statistically different infection rate among type of CVCs in the study cohort. Improving the curriculum with web‐based videos would allow education to be more easily updated. Standardized best practice bundles for caring for children with IF in the home need to be developed similar to current models of care for the pediatric hem/onc population
Gallotto et al^23^	2019	USA	Discharge curriculum and impact on 30‐day readmission rates and CLABSI rate	IF families discharging from IF programs	None	Review discharge curriculum and its impact on 30‐day readmission rates and ambulatory CLABSI rate	Retrospective review of discharge curriculum	CLABSI dec. from 3 to 0.8 per 1000 CVC days 30‐day readmission rates dec. from 40% to 28%	Standardized discharge education can have a positive impact on patient outcomes in the home. Quality metrics need to be developed to benchmark program outcomes and help standardize the transition from hospital to home
Raphael et al^6^	2018	USA	24‐h hands‐on training using home infusion supplies before discharge	Intervention group: 9 families	Control group: 12 families	Primary: assess if a hands‐on, 24‐h, HPN experience impacts 30‐day readmissions and postdischarge events Secondary: parent perception of discharge readiness, quality of discharge teaching, and coping	Prospective nonrandomized trial	No significant difference in discharge readiness and quality of teaching Nonsignificant trend of increased postdischarge coping in intervention group Readmission rates and postdischarge events similar in both groups	All participants had at least one HPN‐related complication after discharge. 30% of the patients were readmitted within 30 days of discharge. There were 16% increased odds for readmission for every additional week of hospitalization with >80 days length of stay being the average for the study cohort. Quality metrics for a HPN discharge process are needed to benchmark and compare methods among centers
Pierik et al^24^	2021	Canada	Video educational series on central line cares	11 out of 14 families in an IF program	2 years retrospective CVC complication data	Assess the impact of an educational video on CVC complications 1 year postdischarge	Pre‐post intervention study	30% of families reported feeling somewhat confident in CVC cares Having at least 1 CVC complication dec. from 75% to 45% Having 2 CVC complications dec. from 63% to 45% Overall, total CVC complications dec. significantly from 7.88 to 2.65 events per 1000 catheter days (*P* = 0.46)	Low‐cost video education can enhance current training programs, increase caregiver confidence, and dec. CVC‐related complications

Abbreviations: 24/7, 24 h, 7 days a week; CLABSI, central line–associated bloodstream infection; CRBSI, catheter‐related bloodstream infection; CVC, central venous catheter; dec., decreased; GI, gastrointestinal; hem/onc, hematology‐oncology; HPN, home parenteral nutrition; IF, intestinal failure; PN, parenteral nutrition; QOL, quality of life; SBS, short bowel syndrome.

**Figure 2:**
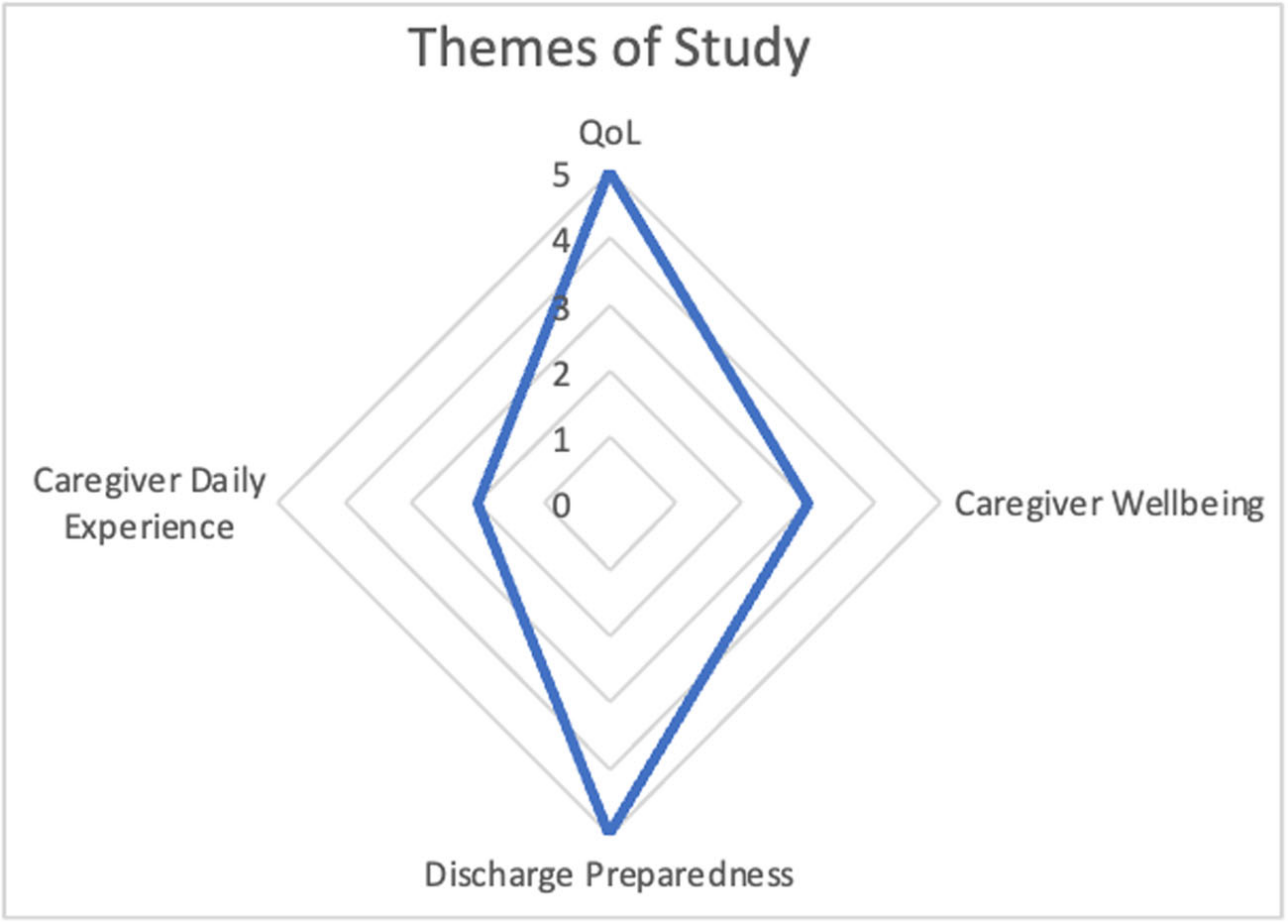
Themes of included studies. QoL, quality of life.

After data abstraction, the studies were grouped by themes identified by MBHD and VBT. The number of studies that focused on each theme is visualized in Figure 2. Of the 15 studies, 5 studies described the caregiver's quality of life (QOL), 5 studies described caregiver's discharge preparedness, 3 studies discussed caregiver well‐being (stress, anxiety, and depression), and 2 studies described the caregiver's daily experience of providing care.

Measures used to assess for QOL and caregiver well‐being varied widely. Three studies used the Hospital Anxiety and Depression Scale.^16^, ^17^, ^18^ Two studies use the PedsQL Family Impact Module.^16^, ^19^ Additional measures were used for individual studies: Parental Stress Index (PSI) Short Form, Pediatric Inventory for Parents, Readiness for Hospital Discharge Survey, Quality of Discharge Teaching Scale, Post‐Discharge Coping Difficulty Scale, *International Classification of Functionality, Disability and Health: Children and Youth Version*, State‐Trait Anxiety Inventory with State Anxiety, Psychological General Well‐Being Index, Italian Multidimensional Test for the Measurement of Self‐Esteem (Test Multidimensionale dell‐Autostima), Distress Thermometer for Parents, health‐related QOL, Generalized Anxiety Disorder 7‐Item, PSI Fourth Edition, and additional unvalidated tools developed by the research teams. None of the measures were developed specifically for caregivers of children with intestinal failure.

### QOL (*n* = 5)

QOL for the caregiver was assessed as it compared with healthy caregiver controls and with caregivers for other chronically ill children in these studies. Researchers in two studies reported that there were no statistical differences in the caregiver QOL for the family caregiver when comparing families with a child with intestinal failure in the home to healthy controls, but families of children with intestinal failure had lower QOL when compared with families of other chronically ill children.^18^, ^20^ In another study, family caregivers of children with intestinal failure had lower QOL when compared with a healthy family cohort, but contrary to other studies, the intestinal failure families had similar QOL scores compared with a cohort of families of other chronically ill children.^19^ In another study, caregivers of children with intestinal failure demonstrated a statistically significant decreased health‐related QOL (*P* < 0.01) when compared with a healthy comparison group.^16^

Factors identified as negatively impacting a caregiver's QOL included chronic sleep deprivation, psychological stressors, inability to take time away with their partners, and a lack of access to caregiving and resource support.^21^, ^22^ Despite the constant demand, caregivers' QOL was less impacted by the logistical management of the child's medical needs and more impacted by chronic lack of sleep and psychological stressors.^21^, ^22^

Other factors were identified as impacting caregivers and their QOL. Children with intestinal failure who also had a comorbid condition and four or more inpatient admissions in the prior year had caregivers with lower caregiver QOL scores.^19^ The lowest QOL scores were found in caregivers of infants and in those with 5–7‐year‐old children with intestinal failure.^19^ In addition, families with children with intestinal failure over 5 years of age reported a lack of information, poor communication, and poor inclusion of the family when interacting with the healthcare team when compared with families with children <5 years old, which negatively impacts QOL.^20^

### Discharge preparedness (*n* = 5)

All researchers identified that standardized discharge instructions before discharge to home can positively impact patient outcomes.^2^, ^3^, ^6^, ^23^, ^24^ Because central line–associated bloodstream infections (CLABSI) are a common cause of readmission for children with intestinal failure, many of the articles related to caregiver discharge preparedness have an emphasis on reducing rates associated with infection. Because the caregiver is primarily responsible for the CVC (and any associated infectious complications in the home), focusing training and interventions on the caregiver was found to decrease CLABSI. One study showed CLABSI rates decreased from 3.0–7.0 to 0.8–2.9 infections per 1000 CVC days (*P* = 0.001) after standardized discharge curriculum was implemented.^3^, ^23^ In another study, the total number of CVC complications were found to significantly decrease from 7.88 to 2.65 events per 1000 catheter days (*P* = 0.046) after implementing an educational video series related to CVC and home infusion care.^24^ Also, most importantly, one study noted the 30‐day readmission rate decreased from 40% to 28% after implementing a standardized five session curriculum.^23^

Several studies assessed effectiveness of in‐hospital training programs and teaching curriculum. Following the use of a 24‐h hands‐on home simulation training program in a hospital setting before discharge, researchers found there was no significant difference in discharge readiness and quality of teaching when compared with a control group that received standardized didactic home parenteral nutrition teaching and hands‐on home infusion pump training by nursing and nutrition staff members.^25^ Low‐cost video education can enhance current training programs, increase caregiver preparedness, and decrease CVC‐related complications.^3^, ^24^

### Caregiver well‐being (*n* = 3)

Caring for a child with intestinal failure leads to a considerable burden of time and energy for the caregivers. Caregivers of children with intestinal failure were noted to have higher levels of stress compared with healthy comparison parents (*P* < 0.01).^16^ In one study, 65% of parents demonstrated elevated anxiety, 35% of whom had anxiety levels above clinical threshold,^17^ with most experiencing medium to high levels of anxiety.^26^, ^27^ Furthermore, increased depression was found in 30% of parents of children with intestinal failure, 15% of which had depression symptom levels above the clinical threshold.^17^ Another study found that when assessing parental partners caring for children on home parenteral nutrition, mothers were typically noted to have more depressive symptoms, whereas fathers reported greater distress with daily activities.^18^

Caregivers of children with intestinal failure also had high levels of stress, anxiety, and depression when compared with other chronically ill pediatric patient populations.^16^, ^17^ Another study found caregivers reported a lack of support from relatives and other care providers.^26^ Complex treatment plans with limited respite care (which provides short‐term relief for primary caregivers) were found to contribute to caregiver anxiety and hypervigilance, which significantly impacted the caregiver's well‐being.^27^

### Caregiver daily experience (*n* = 2)

Upon exploring parents' daily experience of caring for a child with intestinal failure, one study identified five themes: (1) caregiving is a 24‐h, 7‐day commitment; (2) a constant risk of death; (3) difficult feelings and emotions; (4) caring for a child with intestinal failure impacts all aspects of family life; and (5) adapting and functioning as a family is challenging.^28^ Caregivers make significant sacrifices in their personal and family lives to meet the caregiving needs of their child.^28^ Belza and colleagues found that on average, parents spend 29 h per week providing informal care, with the most significant time constraint being the administration of home parenteral nutrition and caring for the CVC.^29^

The identification of resources and support to enable caregivers to provide high‐level care in the home is crucial to allow families to sustain the child's medical needs outside of the hospital setting.^28^ In addition to providing home parenteral nutrition, caregivers are also managing other medical devices such as 41% of families reporting their child also had an enteral tube, which they reported as taking a median of 4.6 h a week to care for, and 38% of families were also managing an ostomy, which was reported as taking 1.3 h per week.^29^ Caregivers reported using a home nurse for a median of 1 h a week with 47% of families reporting 0 h.^29^

To provide care in the home, families reported having to spend a median of $2379.23 a year.^29^ The responsibility of care is tremendous and challenging; therefore, it is difficult for families to feel they can entrust this responsibility with people outside of their family. Alternate caregivers are important partners to decrease care burdens.^28^

## DISCUSSION AND PRACTICE RECOMMENDATIONS

The purpose of this scoping literature review was to explore the experiences of caregivers providing medical care (such as home parenteral nutrition therapy via a CVC) for their child with intestinal failure in the home. This review identified a small number of heterogeneous studies related to the experience of caring for a child with intestinal failure from the lens of the caregiver. Four main themes regarding the caregiver experience were identified from the review: QOL, discharge preparedness, caregiver well‐being, and caregiver daily experience. A summary of the main themes can be found in Table 3.

**Table 3:** Themes and solutions identified from scoping review studies.

Themes identified from studies	Practice gap recommendations
Quality of life	Caregivers of children with intestinal failure have higher levels of anxiety and depression when compared with caregivers of healthy children and caregivers of other chronically ill children^30^, ^31^ Studies had contradicting evidence related to caregiver quality of life, and additional studies should be conducted to assess true caregiver quality of life for this unique patient population^16^, ^18^, ^20^
Discharge preparedness	Standardized discharge curriculum in place at an institutional level^3^, ^23^, ^25^ Provide instructional training to more than one caregiver to learn at an individualized pace appropriate for their learning needs Develop national guidelines and quality benchmarks for intestinal failure program^6^, ^23^
Caregiver well‐being	Periodic structured caregiver assessments in clinical practice should be implemented to screen for anxiety, depression, and stress with referral to subsequent mental health treatments as a part of routine care^17^, ^21^, ^27^ A framework to fulfill mental health needs should be implemented with referral to subsequent mental health treatments as a part of routine care^17^, ^21^, ^27^ Mental health professionals should be added as team members in intestinal failure programs to meet caregiver needs^18^, ^19^, ^20^, ^21^, ^27^
Caregiver daily experience	Caregiving demands for children with intestinal failure requires an average of 29 h a week of informal caregiving (unpaid care provided to a family member or friend^32^) to meet the treatment needs of the child^29^ These hours do not include the constant vigilance required on the part of the caregiver to ensure that medical devices and treatments do not fail

The results indicate that caregivers of children with intestinal failure have higher levels of anxiety and depression when compared with caregivers of healthy children and caregivers of other chronically ill children. It is important to note that caring for children with other chronic conditions do not always equal the caregiving demands for children with intestinal failure, which requires an average of 29 h a week of informal caregiving (unpaid care provided to a family member or friend^32^) to meet the treatment needs of the child.^29^ This quantifiable amount of time does not include the constant need for the caregiver to remain constantly vigilant (24 h a day, 7 days a week) for medical device malfunction, monitoring for sepsis, and other urgent complications associated with caring for the child with intestinal failure. Caregiver QOL is lower when compared with caregivers of other chronically ill populations and is most impacted by the daily aspects of care, including chronic sleep deprivation and psychological stressors.^21^, ^22^

Despite caregivers feeling confident and prepared for discharge following the in‐hospital discharge education and providing the necessary procedures at home, researchers noted that complications related to the CVC and home parenteral nutrition were common.^6^, ^24^ Postdischarge complications such as CVC infection and breakage and the need for readmission may be improved by ensuring standardized discharge curriculum is in place and provide instructional training to more than one caregiver to learn at an individualized pace appropriate for their learning needs. Ensuring training of a second caregiver at time of discharge enables respite care for the primary caregiver.

The findings of this scoping review regarding caregiver QOL are similar to other studies of chronically ill children with conditions such as cancer, asthma, sickle cell disease, diabetes, epilepsy, and cystic fibrosis, which show parental stress contributes to poor psychosocial adjustment for the caregiver and potential poor health outcomes for the child.^30^, ^31^ Studies had contradicting evidence related to caregiver QOL. One study reported that there was a statistically significant decreased health‐related QOL when compared with a healthy comparison group,^16^ whereas two other studies found no difference.^18^, ^20^

Researchers also highlighted the importance of developing a quality metric to benchmark program discharge preparedness and help standardize education and training to aid in the transition from hospital to home.^3^, ^23^, ^25^ The lack of a standardized model of care or quality benchmarks for programs leads to inconsistency in methods to prepare families for discharge or ensuring program quality because several programs have published differing discharge protocols.^6^, ^23^

An opportunity for caregiver support, identified in several studies, include periodic structured caregiver assessments in clinical practice to screen for anxiety, depression, and stress with referral to subsequent mental health treatments as a part of routine care. Routine caregiver screening can mitigate the known increases in stress and depression and avoid complications that can impact patients with intestinal failure themselves.^17^, ^21^, ^27^ Once the caregiver's well‐being is assessed, a framework to fulfill those needs should be implemented. Adding additional team members, such as mental health professionals, can support the caregiver in providing care to the child with intestinal failure in the home.^18^, ^19^, ^20^, ^21^, ^27^

## LIMITATIONS AND IMPLICATIONS FOR FUTURE RESEARCH

Several limitations and gaps were identified in this scoping review. First, all studies included were from single healthcare centers. Second, all studies used a wide variety of measures to assess caregiver stress, depression, anxiety, and QOL, limiting the ability to perform a comparison of the caregiver experience through a meta‐analysis of the results. Third, the intervention studies identified in this scoping review examined improvement of the discharge to home process (four studies total) but did not identify ways to identify caregiver needs.

Future research is needed to identify and evaluate interventions that will effectively support caregivers of children with intestinal failure and address their mental health needs, including increased depression, stress, and anxiety. To help inform future caregiver wellness intervention studies, more qualitative research is needed to provide insight into the perspective of the children with intestinal failure regarding their actual QOL and experiences.

## CONCLUSION

Caregivers of children with intestinal failure receiving home parenteral nutrition experience a high burden of care, which contributes to increased levels of stress, anxiety, and depression. Treatment teams need to identify ways to regularly assess the anxiety, stress, and emotional distress of caregivers and develop interventions to support parents and families. By better understanding the experiences of the caregiver of children with intestinal failure, programs that offer optimal training and support for the caregiver in the home can be developed to optimize the care for the child. Existing literature suggests that improved discharge preparedness can lead to positive patient outcomes, including lower CLABSI, total CVC complications (including occlusion and breakage), and readmission rates. A comprehensive intestinal failure program can not only optimize the child's outcomes but also improve the caregiver QOL.

## AUTHOR CONTRIBUTIONS

**Mary Beth Hovda Davis**: conceptualization; investigation; writing—original draft; methodology; validation; visualization; writing—review and editing; and formal analysis. **Valerie Boebel Toly**: writing—original draft; methodology; validation; writing—review and editing; and formal analysis. **Erin Weber**: writing—original draft; validation; methodology; writing—review and editing; investigation; and formal analysis. **Riad Rahhal**: formal analysis; writing—original draft; writing—review and editing; and methodology. **Ann Marie McCarthy**: conceptualization; writing—original draft; methodology; validation; writing—review and editing; and visualization.

## CONFLICT OF INTEREST STATEMENT

None declared.

## Supporting information

PRISMA‐ScR‐Fillable‐Checklist JPEN.
